# Current agreement between ActiGraph and CUPAR in measuring moderate to vigorous intensity physical activity for adolescents

**DOI:** 10.1186/s12887-024-04541-4

**Published:** 2024-01-20

**Authors:** Yijuan Lu, Liang Hu, Kehong Yu

**Affiliations:** 1 School of Physical Education, Hangzhou Normal University, Zhejiang Province, Hangzhou, 311121 China; 2 Department of Sport and Exercise Sciences, College of Education, Zhejiang University, Hangzhou, 310000 China; 3 A Center for Sports Modernization and Development, Zhejiang University, Hangzhou, 310000 China

**Keywords:** Physical activity, Measurement, ActiGraph, Reliability

## Abstract

The study aims to develop and validate the Curriculum-related Physical Activity Recall questionnaire (CUPAR) as a measure of physical activity in adolescents. 83 middle-school students (13.23 ± 0.74 yrs) completed the CUPAR and whore ActiGraph accelerometers for seven consecutive days. Correlations and Bland–Altman plots were to examine the agreement between these two measures. Significant correlations were observed between the CUPAR and ActiGraph accelerometer for 5-day MPA (*r* = 0.29, *p* < 0.01), and for both 5-day and 7-day VPA (*r* = 0.47 and 0.79, ps < 0.01), and MVPA (*r* = 0.79 and 0.42, ps < 0.01). Plots showed reasonable agreement between the CUPAR and ActiGraph estimates of VPA and MVPA. The agreement between CUPAR and ActiGraph was higher for in-school VPA (*r *= 0.58, *p* < 0.01) and MVPA (*r* = 0.44, *p* < 0.01) as compared to the out-school VPA (*r* = 0.22, *p* < 0.05) and MVPA (*r* = 0.26, *p* < 0.05). The CUPAR can reduce respondents’ burden, representing a reliable and efficient measure of physical activity among adolescents, especially for PA occurred during in-school sessions and at vigorous intensity.

**Supplementary Information:**

The online version contains supplementary material available at 10.1186/s12887-024-04541-4.

## Introduction

Regular physical activity (PA) in adolescents contributes to physical, psychological, and social well-being [1, 2]. However, many children and adolescents fail to meet the WHO recommendation of at least 60 min of moderate to vigorous physical activity (MVPA) per day [3, 4]. Unlike adults, some particular factors such as school environment, peer pressure, and parental support exert significant influence physical activity participation among adolescents [5, 6]. Therefore, measuring physical activity levels plays a key role in understanding patterns and correlates/determinants of PA behavior in adolescents, which are fundamental to designing, implementing and evaluating PA interventions for adolescents [7]. However, such research endeavors are often hindered by the challenge of applying appropriate measures of PA, which often involves the balance of validity, reliability, and convenience [8]. This is particularly important in the adolescent populations give those cognitive limitations and social desirability are prominent factors to consider in the measurement of PA [9]. Students enter junior high school gradually from mechanical literacy to meaningful literacy transformation, in terms of the purpose of memory, conscious memory and intentional reproduction gradually dominate, but overall, still dominated by unconscious memory. Traditional physical activity questionnaires (e.g., IPAQ) require students to recall physical activity from the past week, such as the cumulative number of days and duration of large, medium, and small physical activity intensities. Difficulty in differentiating between middle school students for categorizing physical activities of different intensities. It is difficult to accurately differentiate between low and medium intensity, and medium and high intensity. In addition, the unfocused duration of activities for middle school students, such as 2–3 min of high intensity followed by interruptions and multiple times a day. Asking middle school students to recall the cumulative number of times and times of the week is difficult.

Objective measurement (e.g., indirect calorimetry) are considered accurate and valid methods for measuring total energy expenditure [10, 11]. However, most objective methods are high on the continuum of accuracy but low in the practicality continuum. With the advantages of low cost and subject burden, self-reported measures (e.g., PA questionnaire, PA recall records and logs) are still desirable for its convenience and low expenses, especially when large-scale PA data need to be collected among adolescents [9]. However, greater complexity is associated with lower reliability of a PA questionnaire [12]. Simplifying a PA measure without sacrificing its validity is thus an ongoing and important matter.

Questionnaires designed in the form of PA recall have been embraced by many researchers for its low-cost and adequate validity. However, the somewhat cumbersome process of recalling PA information throughout the day still hinders its practical application. Considering that factors such as cognitive limitations and social desirability may lead to recall bias and hinder the accuracy of PA measurement among adolescents [9], a number of retrospective PA record/logs were designed for subjects to record their activities in segmented time periods, so as to help them recall their past behaviors accurately. For example, the Previous Day Physical Activity Recall (PDPAR) [7], a self-report instrument that was designed specifically for children and adolescents, segments the previous days into 30-min time blocks. This approach provides a given timeframe so that the respondents can more easily recall their activity information, resulting in smaller recall bias than questionnaires, and satisfactory reliability and validity [13–16]. However, a prominent disadvantage is that it is often considered burdensome for adolescents.

To this end, a careful examination of the nature of adolescents’ daily activities would suggest a possible means of reducing answering burdens of self-reported PA logs. In fact, activity patterns of most adolescents are highly structured during school hours of weekdays. Therefore, we propose a new tool, namely the Curriculum-related Physical Activity Recall Questionnaire (CUPAR), for measuring PA among adolescents by fully taking advantage of the structured nature of their curriculum. In essence, the approach simplifies the process of PA Recall by assessing the minutes of moderate physical activity (MPA), vigorous physical activity (VPA) and MVPA per day during school time using curriculum information collected from the teachers, without creating burdens on the students. In this way, the respondents only need to recall the PA that occur outside of school hours. The questionnaire has an origin in traditional PDPAR, but consists of two parts: structured in-school part and unstructured out of school part. The in-school PA is based on curriculum information of each student, which is mostly the same for a given class of students. The organization of out of school PA measurement remain the same as PDPAR, asking the students to recall the type and intensity of activities in each 30-min block.

Therefore, the purpose of this study is to develop the CUPAR as a curriculum-related PA measure for adolescents on the basis of PDPAR, and test its criterion validity using accelerometer as the criterion measure. Presumably, by introducing the concept of curriculum-based PA assessment, the re-structured Physical Activity Recall has several advantages: 1) it simplifies the process of answering the survey, and substantially reduces the burden on the part of students; 2) it enables researchers to easily assess in-school and out of school PA respectively, and separately calculates the MPA, VPA, and MVPA per day. By so doing, the CUPAR has the potential of becoming a reasonably convenient and reliable tool for measuring PA among adolescent students. The following two hypotheses were formulated: 1) there is a significant correlation between PA (calculated as minutes of MPA, VPA and MVPA per day) measured by CUPAR and accelerometer; 2) the association between minutes per day measured by CUPAR and accelerometer is greater in the in-school part than that of out of school time.

## Materials and Methods

### Sample

Data were collected from 83 students (13.23 ± 0.74 yrs) in a middle school in Hangzhou, Zhejiang Province, China. With the help of Physical Education (PE) teacher, students were recruited in the spring term (April 2019) from PE classes. A total of 86 students (43 boys, 43 girls) were invited to participate the study. All of them returned signed informed consents from their parents or guardian. The study procedures were approved by the research ethics board of Zhejiang University (No.2020–002, 2020.07.22). The participants received a small gift (e.g., Notebook, Fountain Pens) for their participation.

### Measures

#### Curriculum – related Physical Activity Recall(CUPAR)

The design of CUPAR was produced by four researchers after several group discussions. According to the CUPAR design, every day was divided into 20 in-school time blocks and 14 out of school time blocks for weekdays, and 36-time blocks for weekends. For each time block, minutes of MPA, VPA and MVPA per day were calculated by summing up the durations of each activity reported in all the time blocks. The translated English version can be downloaded from the supplementary files.

To further improve the quality of the data recorded, the tool provides a numbered list of common activities with reference to the PDPAR in conjunction with the activities in which students typically engage. The list consists of 40 entries in five categories: at home, diet, transportation, at school and exercise. In addition, fill-in-the-blanks (item 41) are added for students to fill in other physical activities that are not listed. The Youth Compendium of Physical Activities [17] was used to determine the in-tensity category of PA in this study. This code covers 244 specific physical activities, and the common activities selected for the tools can be found in the code. As described previously, the central concept of the CUPAR is to simplify the process of collecting information (i.e., type and duration) of activities occurred in each class by consulting school teachers.

Specifically, the CUPAR is divided into organized and unorganized forms. The organized part is mainly in school part, including curriculum, organized exercise recess and morning exercise; The rest are unorganized parts, including free play, extracurricular activities and lunch break of before school, after school and in school part. For the organized part, researchers collect curriculum information by consulting teachers in the target school. Among them, physical education curriculum is assigned according to the arrangement of classroom content and exercise load. Because Chinese students’ learning activity at classroom with a sit posture, Therefore, the default of classroom curriculum is sedentary behavior. The morning exercise and the organized exercise recess are assigned according to the exercise content given by the physical education teacher. The morning exercise is a 20 min moderate to high intensity running exercise, and the organized exercise recess is a 30 min light to moderate intensity radio exercise. Students only need to recall and fill in the unorganized part, including activities during a number of 10-min breaks between classes by choosing one of the four options: A—sitting and chatting, reading or doing homework; B – walk around; C—unstructured indoor play; D—unstructured outdoor play; the transportation means between school and home, etc.

In the out of school part, students recall their activity in every 30-min block. Similar with PDPAR, students were instructed to write a code of activity in each block based on a list of activity and corresponding code, and to rate the intensity level (light, moderate, or vigorous intensity), a picture illustration is provided to help the students understand how to accurate rate intensity. For mins of MPA, VPA and MVPA per day calculation, a MET value was assigned to each 30-min block based on the type of activity described, the intensity level checked by the student, and the compendium of PA [17, 18]. Numbers of 30-min blocks in moderate (3.0–5.9 METs), vigorous (≥ 6 METs), and moderate-vigorous (≥ 3 METs) activity categories were summed respectively, and multiplied by 30 min to generate the total durations of each intensity category.

#### Accelerometry

Objective assessment of PA for 7 days (five weekdays and two weekend days) was performed using the ActiGraph accelerometer wGT3X-BT model accelerometer (Pensacola, FL, USA). It is a tri-axial accelerometer designed to acceleration ranging in magnitude from 19 g with a frequency response of 30 Hz [9]. Movement counts are averaged over a defined epoch (1 s in the current study), and the data are stored in memory and downloaded to a computer. Students wore the ActiGraph accelerometer using an elastic waist belt that help the accelerometer to be placed over the right waist. Previous studies in children and adolescents have supported the validity of the ActiGraph accelerometer under laboratory [19, 20] and free-living conditions [21]. This research will include 3 valid weekdays and 1valid weekend day. A weekday or weekend test day is defined as a test day with at least 10 valid wear hours. with the valid wear hours to include 40 min or more of non-zero accelerometer data.

### Procedure

All students completed the CUPAR every day, during which they wore the ActiGraph accelerometer for seven consecutive days, 24 h per day. Trained staff placed the accelerometer directly on students’ right waist during school on a Monday and removed them on the following Monday. Then the students were instructed in small groups on monitor use and told to remove the ActiGraph accelerometer only for bathing or swimming.

Students received instructions on the CUPAR including written examples and student completion of a practice diary, which informed them how to record their activities on CUPAR at the end of each day. A one-page sample diary was provided as an example, along with a list of common activities and numerical codes used by Weston et al. [22].

### ActiGraph accelerometer data processing

The raw ActiGraph accelerometer data for each subject was downloaded to a computer using the ActiLife Software. All days with less than 10 h of recorded time were excluded from analysis. The age-specific count ranges developed by Evenson Children (2008) [23], were used to determine the number of minutes of moderate and vigorous activity (see Table 1).

**Table 1:** Count Range for the ActiGraph Accelerometer by Intensity

Activity Level	Evenson Children (2008) (Count/min)
Resting/Light (< 3 METs)	0–2295
Moderate (3–5.99 METs)	2296–4011
Vigorous (≥ 6 METs)	≥ 4012

### Statistical analysis

Data processing and analysis were performed by two types of software. First, IBM SPSS Statistic 25 (IBM, Armonk, NY, USA) was applied to: 1) calculate the Spearman rank order correlations to examine the association of average minutes of MPA, VPA, and MVPA collected by the CUPAR diary and ActiGraph accelerometer; 2) Paired t-tests were used to examine the difference between paired means. To determine if the agreement between CUPAR and ActiGraph accelerometer is acceptable, we refer to Cohen's criterion of correlation coefficients [24], such that a *r* value between 0.00-0.09 represents no effect, whereas 0.10-0.29, 0.30-0.49, above 0.50 represent small, moderate and large effect respectively.

Second, Bland–Altman plots were completed using the MedCalc 15.8 software to assess the agreement between the CUPAR and ActiGraph accelerometer (average activity over 7 d). The 95% limits of agreement (LoA) were used for describing the total errors between the methods. The final consistency evaluation of the two methods was conducted by correlation analysis and Bland-Altman plot. The Bland-Altman plot [25], or difference plot, is to examine the agreement between the methods. The differences (or alternatively the ratios) between the methods are plotted against the averages of them. Alternatively [26] the differences can be plotted against one of the two methods, if this method is a reference or "gold standard" method.

When the measurement results are expressed in quantitative data, the issue of consistency evaluation is worth considering. The commonly used methods in clinical work are paired t test, correlation coefficients, regression analysis, and so on. However, these analysis techniques may lead to inconsistency of two different methods [27]. A strength of the current study involves the comprehensive examination of centralized trend, discrete trend and correlation, which are key components of validity evaluation. The Bland-Altman plot [28, 29], or difference plot, is a graphical method to compare two measurements techniques. In this graphical method, the differences (or alternatively the ratios) between the two techniques are plotted against the averages of the two techniques [30]. Alternatively, the differences can be plotted against one of the two methods, if this method is a reference or "gold standard" method. Only when three aspects, namely correlation, centrality and dispersed trend, are identical, "consistency" can be achieved and the "interchangeability" between methods be explained [31]. In this study, we consider these three aspects and criterion validity of the CUPAR was consistently supported both by correlation analysis and Bland-Altman plot.

## Results

Eighty six students initially participated in the study, 3 boys (3.5%) were excluded from the analysis due to incomplete data either in ActiGraph accelerometer or CUPAR. Using the algorithm, in the first step, students who do not satisfy the effective number of days of wear and the effective time of wear per day are excluded. In the second step, the total valid wearing time is divided by the number of valid wearing days to get the average daily valid wearing time. Hence, 40 (48.2%) boys and 43 (51.8%) girls were included in the final analysis. Subject characteristics of the final sample (*n*=83) are shown in Table 2.

**Table 2:** Characteristics of Study Participants

	Boys	Girls	Total
N (%)	40(48.2)	43(51.8)	83(100)
Age (yr)	13.18 ± 0.75	13.28 ± 0.73	13.23 ± 0.74
Height (cm)	161.62 ± 6.58	157.0 ± 4.35	159.23 ± 8.01
Weight (kg)	51.63 ± 10.73	48.63 ± 9.01	50.07 ± 9.92
BMI (kg/m2)	19.67 ± 3.46	19.70 ± 3.44	19.68 ± 3.43
Daily wear time (min)	813 ± 57	805 ± 55	809 ± 55

Values are presented as mean ± SD. *BMI* Body Mass Index

Spearman correlation analysis was conducted to examine the agreement between CUPAR and the ActiGraph accelerometer data and the results were shown in Table 3. During the 5 weekdays, significant correlations were observed between the CUPAR and ActiGraph for MPA, VPA and MVPA (rs = 0.29 – 0.79, *p* < 0.01). When the entire 7 days are concerned, significant correlations between the two measures were found for VPA (*r* = 0.40, *p* < 0.01) and MVPA (*r* = 0.42, *p* < 0.01), but not for MPA (*r* = 0.19, *p*>0.05). Likewise, correlations of in-school VPA (r = 0.58, p < 0.01) and MVPA (*r* = 0.44, *p* < 0.01) were significant and high, but is low and non-significant for out of school MPA, both on weekdays (*r* = 0.16, *p*>0.05) and weekends (*r* = 0.14, *p*>0.05). However, on weekends, the CUPAR measure corresponds reasonably well with ActiGraph data for both VPA (*r* = 0.30, *p* < 0.01) and MVPA (*r* = 0.30, *p* < 0.01). In general, moderate to large correlations were identified between PA measured by the CUPAR and ActiGraph, such an agreement is greater for VPA in comparison with MPA, and, greater in the in-school setting than in the out of school setting.

**Table 3:** Spearman Correlations between the CUPAR and the ActiGraph

Item	PA	In-school			Out-school (Weekday)			Out-school (weekend)			All-day (weekday)			All-day (one week)		
		MPA	VPA	MVPA	MPA	VPA	MVPA	MPA	VPA	MVPA	MPA	VPA	MVPA	MPA	VPA	MVPA
In-school	MPA	0.21	0.474**	0.322**	-0.005	0.012	0	-0.213	-0.205	-0.214	0.134	0.407**	0.699**	0.077	0.307**	0.184
	VPA	0.383**	0.576**	0.498**	0.016	0.001	0.013	0.003	0.014	0.008	0.291**	0.482**	0.634**	0.222*	0.423**	0.375**
	MVPA	0.314**	0.594**	0.442**	0.025	0.017	0.024	-0.118	-0.104	-0.117	0.229*	0.513**	0.801**	0.163	0.422**	0.319**
Out-school (weekday)	MPA	0.294**	0.105	0.268*	0.160	0.144	0.155	-0.031	0.035	-0.008	0.284**	0.14	0.309**	0.118	0.115	0.135
	VPA	0.239*	0.321**	0.327**	0.134	0.219*	0.154	0.054	0.121	0.087	0.231*	0.336**	0.296**	0.137	0.273*	0.268*
	MVPA	0.354**	0.329**	0.406**	0.251*	0.272*	0.261*	0.008	0.121	0.063	0.362**	0.381**	0.468**	0.212	0.342**	0.333**
Out-school (weekend)	MPA	0.107	0.039	0.09	-0.047	0.099	0.004	0.143	0.209	0.163	0.072	0.064	0.082	0.123	0.136	0.131
	VPA	0.02	0.003	0.059	0.097	0.123	0.139	0.296**	0.302**	0.307**	0.061	0.039	0.022	0.119	0.096	0.145
	MVPA	0.161	0.064	0.159	0.073	0.174	0.121	0.262*	0.342**	0.295**	0.157	0.094	0.111	0.220*	0.182	0.217*
All-day (weekday)	MPA	0.328**	0.409**	0.400**	0.157	0.151	0.159	-0.179	-0.117	-0.155	0.291**	0.411**	0.704**	0.157	0.315**	0.248*
	VPA	0.347**	0.524**	0.462**	0.068	0.099	0.076	0.008	0.035	0.017	0.281*	0.469**	0.612**	0.196	0.391**	0.343**
	MVPA	0.401**	0.550**	0.509**	0.199	0.199	0.202	-0.059	0.017	-0.029	0.367**	0.551**	0.794**	0.242*	0.465**	0.395**
All-day (one week)	MPA	0.288**	0.331**	0.333**	0.034	0.18	0.086	-0.071	0.024	-0.037	0.226*	0.350**	0.494**	0.186	0.325**	0.268*
	VPA	0.303**	0.450**	0.417**	0.106	0.204	0.151	0.096	0.108	0.099	0.260*	0.442**	0.560**	0.2	0.402**	0.353**
	MVPA	0.393**	0.461**	0.475**	0.127	0.275*	0.187	0.036	0.118	0.068	0.346**	0.497**	0.623**	0.280*	0.472**	0.419**

*MPA *Moderate physical activity, *VPA* Vigorous physical activity, *MVPA* Moderate-to-vigorous physical activity. *P < 0.05; **P < 0.01

**Fig. 1:**
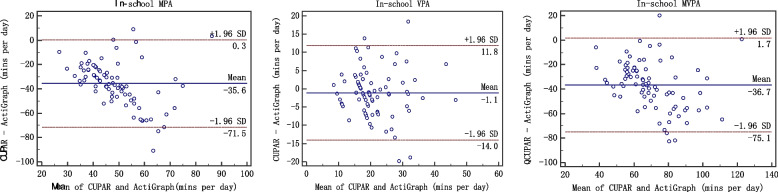
Bland–Altman Plot for Agreement between the in-school CUPAR Diary and the ActiGraph Accelerometer

**Fig. 2:**
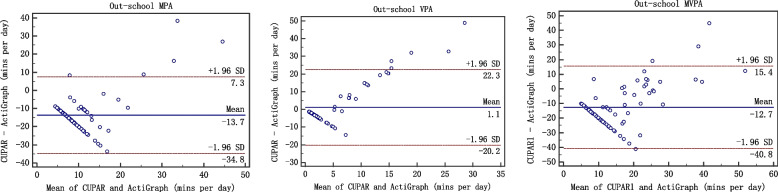
Bland–Altman Plot for Agreement between the out-school (weekday) CUPAR Diary and the ActiGraph Accelerometer

**Fig. 3:**
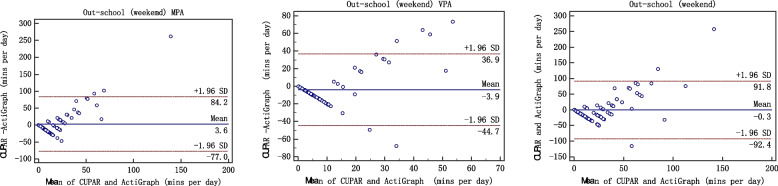
Bland–Altman Plot for Agreement between the out-school (weekend) CUPAR Diary and the ActiGraph Accelerometer

**Fig. 4:**
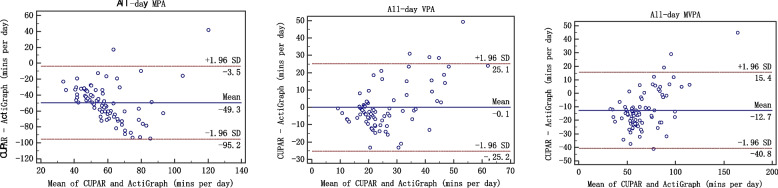
Bland–Altman Plot for Agreement between the all-day (weekday) CUPAR Diary and the ActiGraph Accelerometer

**Fig. 5:**
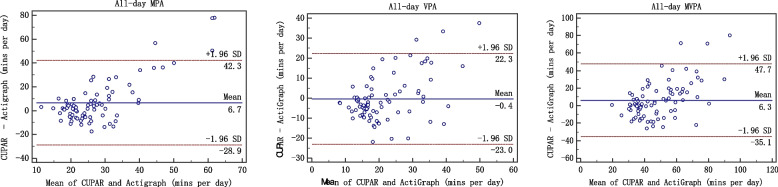
Bland–Altman Plot for Agreement between the all-day (one week) CUPAR Diary and the ActiGraph Accelerometer

Such findings were verified by Bland-Altman plot (figures 1, 2, 3, 4 and 5), which showed reasonable agreement between the CUPAR and ActiGraph on estimates of MPA, VPA and MVPA. The mean difference and the limit of agreement (LoA) between the CUPAR and ActiGraph accelerometer measures for MPA, VPA and MVPA were shown in Table 4 and Figures 1, 2, 3, 4 and 5. Minutes spent on moderate intensity category measured by CUPAR were consistently lower than ActiGraph accelerometer during weekdays, including both the in-school (t = 17.7, *p*<0.01) and out-school (t = 11.6, *p*<0.01) portions, but were higher (t = 3.35, *p*<0.01) in the all-day (one week) part.

**Table 4:** The Mean and Standard deviation (SD), Lower Limit of Agreement (mean-1.96*SD) and Upper Limit of Agreement (mean + 1.96*SD)

		CUPAR		ActiGraph					95% CI of LoA			95% CI of LoA	
	PAL	Mean	SD	Mean	SD	t	P	Lower limit	Lower	Upper	Upper limit	Lower	Upper
In-school	MPA	30.93	10.89	66.53	16.98	-17.70	< 0.01	-71.51	-78.38	-64.65	0.31	-6.55	7.18
	VPA	20.58	7.72	21.69	8.13	-1.54	0.13	-14.00	-16.46	-11.53	11.78	9.31	14.24
	MVPA	51.51	15.70	88.22	21.66	-17.07	< 0.01	-75.11	-82.45	-67.77	1.69	-5.65	9.03
Out-school (weekday)	MPA	3.83	10.43	17.58	5.84	-11.64	< 0.01	-34.84	-38.87	-30.81	7.34	3.31	11.37
	VPA	5.17	10.81	4.11	2.28	0.89	0.38	-20.22	-24.29	-16.15	22.34	18.27	26.41
	MVPA	9.00	14.68	21.69	7.50	-8.06	< 0.01	-40.82	-46.20	-35.44	15.44	10.06	20.82
Out-school (weekend)	MPA	20.42	40.28	16.82	10.07	0.80	0.43	-77.03	-92.44	-61.61	84.24	68.82	99.65
	VPA	8.31	19.91	12.22	10.31	-1.71	0.09	-44.72	-52.52	-36.92	36.91	29.11	44.72
	MVPA	28.74	45.80	29.04	19.44	-0.06	0.95	-92.39	109.99	-74.79	91.79	74.19	109.39
All-day (weekday)	MPA	34.76	17.60	73.20	17.12	-19.22	< 0.01	-95.19	-103.95	-86.43	-3.51	-12.27	5.26
	VPA	25.75	15.11	25.80	8.78	-0.04	0.97	-25.24	-30.05	-20.42	25.14	20.32	29.95
	MVPA	60.51	25.20	84.11	20.38	-8.19	< 0.01	-40.82	-46.20	-35.44	15.44	10.06	20.82
All-day (one week)	MPA	30.60	17.98	23.92	6.48	3.35	< 0.01	-28.92	-35.73	-22.12	42.28	35.48	49.09
	VPA	21.28	12.64	21.65	7.81	-0.29	0.78	-23.04	-27.38	-18.71	22.31	17.98	26.65
	MVPA	51.89	22.96	45.57	12.90	2.73	0.01	-35.06	-42.97	-27.15	47.69	39.78	55.60

*MPA* Moderate physical activity, *VPA* Vigorous physical activity, *MVPA* Moderate-to-vigorous physical activity

The Bland-Altman Plot for Agreement (see Figures 1, 2, 3, 4 and 5) suggest that the majority of dots are within the 95% LoA range, further confirming the results of correlation analysis. That is, CUPAR measures correspond reasonably well with ActiGraph data, especially with regard to the physical activities that occur during in-school time and in the high-intensity category.

## Discussion

In a group of middle-school students, the currents study supported the criterion validity of the CUPAR, a newly designed form of PA recall that significantly reduces adolescents’ answering burdens by assessing PA information during school hours based on curriculums information provided by teachers. As hypothesized, the CUPAR demonstrated moderate validity as indicated by its correspondence with Accelerometer measure, that the CUPAR can be used to quickly and reliably measure PA in studies involving adolescents, especially for assessing in-school PA.

A prominent advantage of CUPAR is that most of the burden of describing in-school PA information was reduced for students, because it is relatively easier for researchers to work with teachers to retrieve that, as compared with traditional means of asking students for the answers, which often involves working with both teachers and students. Given the fact that, unlike adult populations, most students’ activity pattern during school hours is highly structured and consistent in one class/grade, it is unnecessary for each student to go through the whole process of recalling all activity information of each hour during school, as the PDPAR does. According to feedback from daily follow-up researchers, students need 1–2 min to fill out their daily records. Randomly conducting unstructured interviews with students, it was found that 95% of them believed that filling out questionnaires every day for 7 consecutive days did not cause them any time constraints. However, the CUPAR also considers the variations in PA accumulated during each 10-min break between classes. In this regard, the CUPAR is not just a revision of PDPAR. To our knowledge, it is the first PA measure that factor the concept of curriculum into PAR questionnaire design, which fully takes advantage of the structured and consistent nature of curriculums in middle schools. In this way, the in-school part of PA information collection becomes simple, which should motivate the students to better use their time to provide accurate information on items that are not bound to curriculums. This may partly explain the substantial correspondence between CUPAR and ActiGraph data. As can been seen, there are moderate to large correlations between self-reported VPA and MVPA from the CUPAR and the ActiGraph accelerometer for both in-school portion and all-day composite data (ranged from 0.42 to 0.66). This is higher than the previously reported correlations between the PDPAR and Accelerometer in adolescents [32, 33], as well as other studies in youth that have compared other self-report PA recall measures with objective PA measures [33–35].

It should be noted that, the degree of agreements between the CUPAR and Accelerometer varies in different settings. Specifically, the VPA and MVPA measured by CUPAR correspond much better with Accelerometer in the in-school setting than in out of school (weekday) setting. It appears that the CUPAR provides a good estimate of time spent in vigorous PA in reference to ActiGraph data. However, when MPA is concerned, the correlations between the CUPAR and Accelerometer for in-school and out of school PA are both small and non-significant. Such findings suggest that middle-school students may more likely misperceive MPA than VPA. It is relatively easier for students to determine VPA and LPA. However, it is more difficult for them to differentiate MPA from LPA and/or VPA. The students may classify LPA as MPA. As suggested by the analyses, students tend to overestimate MPA in the CUPAR, as compared with MPA measured by ActiGraph.

The study is not without limitations. First, the design CUPAR is highly dependent on curriculums of the target sample. It is important to note that the data collection methods of in-school and out of school parts are substantially different. The agreements between CUPAR and accelerometry of the out-of-school part is less satisfactory than in-school part. Future researchers are encouraged to note the difference and consider interpreting the physical activity data collected from in-school and out-of-school settings in different manner. Second, it should be noted that substantial variations can exist across different schools in terms of class schedules, duration of classes and class breaks. Specifically, in current study, we elected to collect in-school activity information of each class by 40 min and that of class breaks by 10 min, whereas the out-of-school activity were measured in segments of 30 min. Such a timeframe is in accordance with standard procedure set by local educational authorities, which applies to most middle schools in China. However, one should be cognizant that, although such an approach is necessary to reflect the nature of students’ activity patterns, this could be potentially confusing. Not to mention, the duration of each class and class break may vary across different regions and countries. Hence, the CUPAR designed and validated in the current study represents a novel framework of collecting in-school physical activity data in adolescents. In practice, the CUPAR is subject to modifications in terms of durations of class and class break based on the actual curriculum design of each school. Specifically, the curriculum needs to be scrutinized and the scale may be adjusted according to the actual schedule of different schools, or even different grades. It may cause some inconvenience for the researcher. However, considering the substantial reduction of subject burden, such efforts are worthwhile. In addition, with the increasingly wide acceptance of portable electronic devices (i.e., ipad, smart phones), computer applications can be programmed to enable the researcher easily adjust the CUPAR in accordance with the actual schedule of target schools.

Third, as previous noted, we split physical education classes into 10 min of VPA and 20 min of MPA according to the standard procedure set by educational authorities. In practical setting, the teachers can ensure the time spent on different types of physical activity that were designed to be performed in different intensity (i.e., light, moderate and vigorous). However, it could be challenging for the teachers to precisely monitor such intensities. In fact, such an issue represents a longstanding challenge to assess the actual activity level of physical education classes, given that the infrastructure of physical education classes can vary in different schools. In the current study, our approach of interpreting physical education classes appears to be reasonable given the agreements of in-school CUPAR and ActiGraph accelerometer is satisfactory. However, alternative approaches may be considered. For example, as suggested by the 3-day Physical Activity Recall instrument [36], physical education classes can be assigned a literature-based MET value according to the self-reported intensity (light, moderate, hard, very hard) [37]. By so doing, future researchers can adopt the design of CUPAR and simply add the assessment of self-rated intensity of physical education class [36] to better address the above-mentioned issue, and potentially improve the validity of CUPAR.

In the measurement of MVPA time in physical education class, the result of questionnaire measurement is larger than that of accelerometer. In this study, the time of MVPA measured by accelerometer is 8.03 min, which is similar to the existing research in China. For example, Jing Bo [38] pointed out in his research that the duration of moderate to high intensity physical activity of junior high school students is 7.14 min [39] in the conventional teaching of 40 min physical education, while in the questionnaire measurement, the duration of MVPA given by physical education teachers is about 30 min. The reason is that the cut point algorithm of different accelerometers will affect the time of MVPA. The cut point of this study uses Evenson children (2008) algorithm. In the validity test, the content of MVPA tested by Evenson is mainly aerobic sports such as stair climbing, dribble basketball, brisk walk, bicycling, jumping jacks (jumping jacks at 126 bpm with the metronome) and running (running at 4 mph on treadmill).

However, due to the influence of the High school physical education entrance examination, most of the physical education curriculum exercises in junior high schools in China are carried out around the items of high school entrance examination (Long jump, sit up for female, pull up for male, 800 m run for female, 1000 m run for male). After the warm-up run, students are arranged to carry out physical exercises such as high leg lifting, push-up, car pushing, sit ups, frog leaping, step running, etc., and the teaching of movement skills is interspersed with examination items. The majority of force items may be the cause of underestimation of accelerometer measurement.

## Conclusions

In general, the CUPAR can be considered as a simple, time-saving Physical Activity Recall (PAR) measure for adolescents. Although the current study designed and tested the CUPAR among in Chinese middle school students, the questionnaire has potential to be widely used in students of other ages (i.e., primary schools) and other countries, as most adolescents spend the majority of their school time in group-based and structured manner. More future studies are certainly warranted to further test the validity of the CUPAR in students of various ages and countries, and use of more criterion’s measures of PA (i.e., double-labelled water, direct calorimetry) are encouraged in these validity studies. The collective work may help researchers to explore strategies to further improve the convenience (e.g., introducing computer programs to match respondents with curriculum) and validity (especially the low agreement between CUPAR and ActiGraph in MPA) of the CUPAR. 

### Supplementary Information

**Additional file 1. **

## Data Availability

The datasets used and/or analyzed during the current study are available from the corresponding author upon reasonable request.
