# Supplementary material for: Current agreement between ActiGraph and CUPAR in measuring moderate to vigorous intensity physical activity for adolescents
Source: BMC Pediatr. 2024 Jan 20;24:63. doi: 10.1186/s12887-024-04541-4 (PMC10799407; doi:10.1186/s12887-024-04541-4)
Supplement: Supplementary file 1 — Additional file 1. [file 12887_2024_4541_MOESM1_ESM.docx]

**Supplementary file: Curriculum-related Physical Activity Recall questionnaire**

*The following are the specifics of the Physical Activity Recall Questionnaire Related to the Curriculum developed for this study.*

CUPAR

On this page, you will see several pictures. You can choose how you feel when you are engaged in physical activities according to the contents of the pictures.

*一、Sedentary Behavior (SB)：No activity or slight activity, accompanied by slow breathing (reading, eating, washing).*
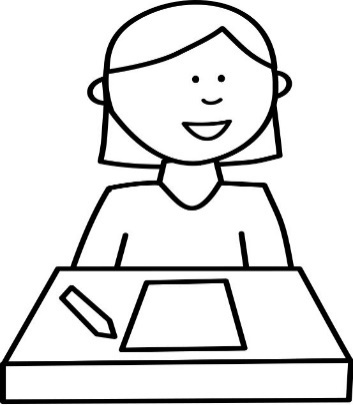

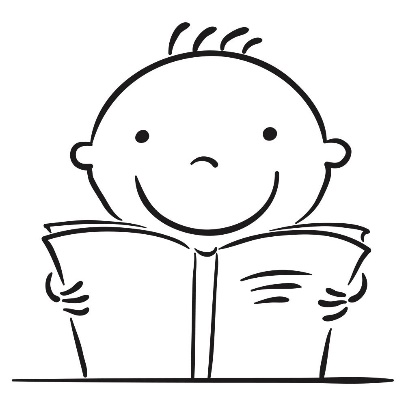

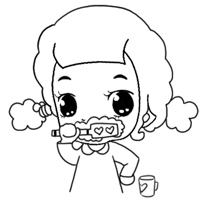

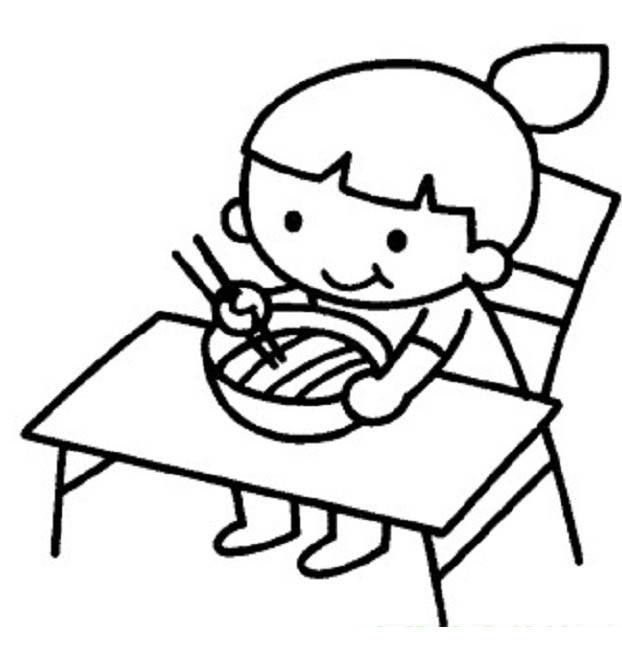


*二、Light：There are some activities that can breathe normally (walking, doing radio exercises).*


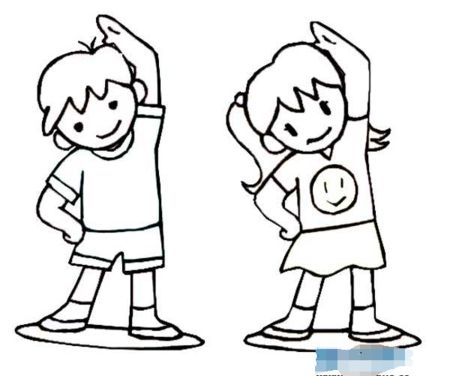

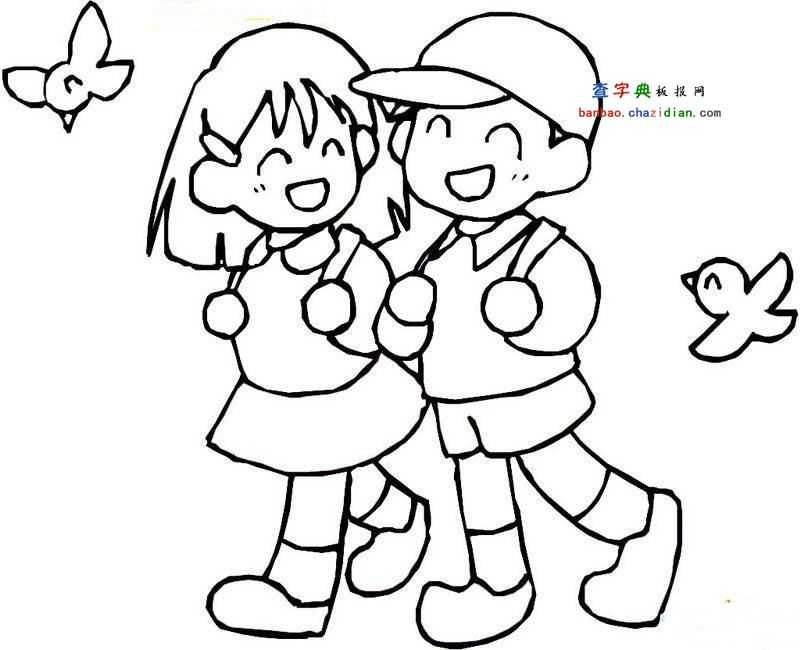

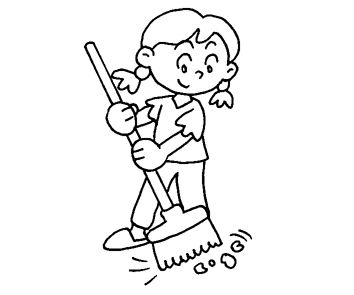


*三、Moderate：Moderate intensity of activity, breathing significantly accelerated (kicking, dancing, weight-bearing exercises).*


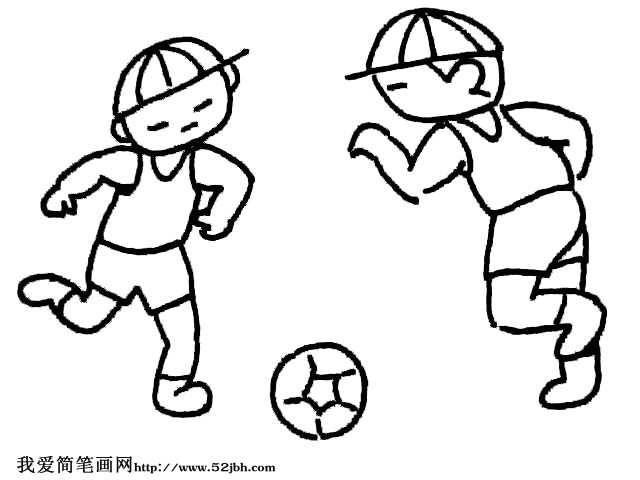

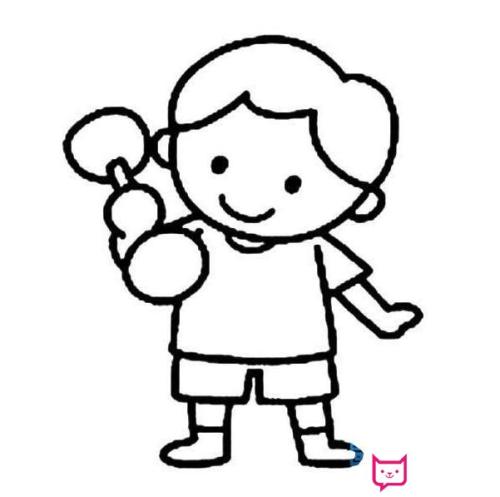

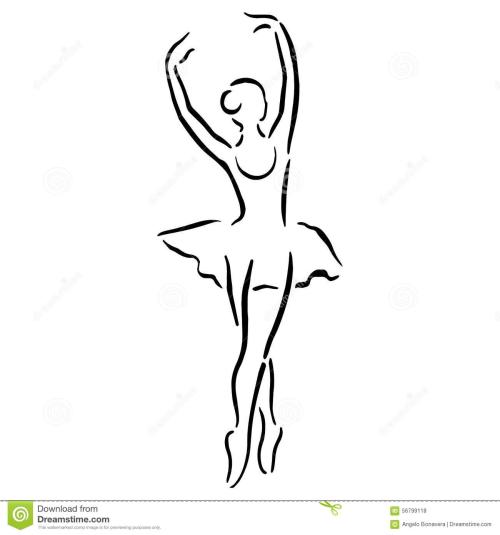


*四、Vigorous：Intense exercise, shortness of breath (fast running, cycling, playing badminton, tennis).*


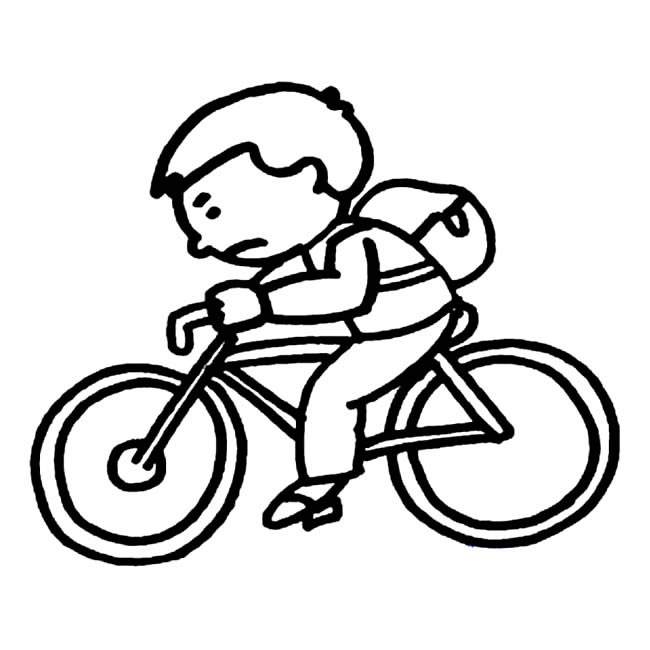

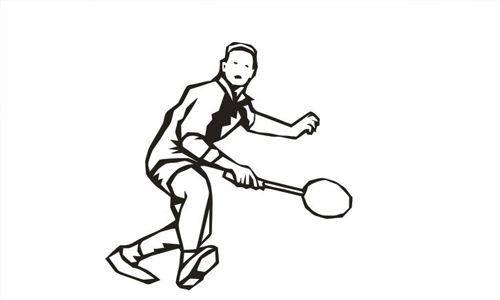

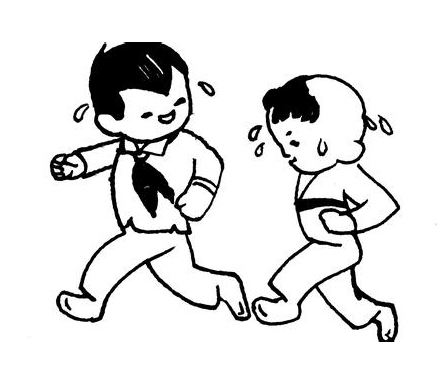


On this page you will see a list of activities, each with its own number.

Note: *1. If the activity you are engaged in is not on the list, you can choose (other) to fill in the activity you have carried out.*

*2. For each time period, write down the number that represents the most important activity you're engaged in at a certain stage.*

*3. Then tick "√" under the form of how you feel when you are engaged in the activity.*

| Time segments | Number | Activity | Time segments | Number | Activity |
| --- | --- | --- | --- | --- | --- |
| At home | 1 | sleep | Physical Activities and sports | 21 | brisk walking |
|  | 2 | dress, wash (comb hair and make-up) |  | 22 | running, jogging |
|  | 3 | take a shower |  | 23 | calisthenics |
|  | 4 | homework |  | 24 | aerobics |
|  | 5 | listen to the music |  | 25 | gymnastics |
|  | 6 | video games, surfing |  | 26 | basketball |
|  | 7 | read |  | 27 | football |
|  | 8 | watching TV or movies |  | 28 | volleyball |
|  | 9 | give sb. a call |  | 29 | badminton |
|  | 10 | housework |  | 30 | table Tennis |
| Diet | 11 | have a meal |  | 31 | tennis |
|  | 12 | eat snacks between meals |  | 32 | roller-skating |
| Transportation | 13 | cycling |  | 33 | swimming |
|  | 14 | by car or bus |  | 34 | martial art |
|  | 15 | walk |  | 35 | wrestling |
| At school | 16 | attend class |  | 36 | chase games |
|  | 17 | music lessons, playing musical instruments |  | 37 | bodybuilding |
|  | 18 | PE class |  | 38 | rope skipping |
|  | 19 | lunch, self-study |  | 39 | yoga stretching |
|  | 20 | free activities |  | 40 | shopping |
| 41 | | Others (mark specific activities) | | | |

**Weekday**

Do you have illness or other special things that hinder your daily physical activities today?

□ A: No

□ B: yes，Please write it clearly

| ***schedule*** | ***time*** | ***activity*** | | | | | | |
| --- | --- | --- | --- | --- | --- | --- | --- | --- |
| Get up： : | | | | | | | | |
| Transportation | □A cycling □B by bus □C walk □D Parents' transfer | | | | | | | |
| Do you participate in morning jogging | | | | | □A Yes □B No | | | |
| Recess 1  8:40-8:45 | □A sitting and chatting, reading or doing homework □B walk around (e.g., Go to toilet)  □C active play (indoor) □D active play (outdoor) | | | | | | | |
| Recess 2  10:40-10:50 | □A sitting and chatting, reading or doing homework □B walk around (e.g., Go to toilet)  □C active play (indoor) □D active play (outdoor) | | | | | | | |
| Lunch  11:35-12:30 | □A sitting and chatting, reading or doing homework □B walk around (e.g., Go to toilet)  □C active play (indoor) □D active play (outdoor) | | | | | | | |
| Noon break (12:30-13:00) | | □A Take a nap □B Reading or doing homework | | | | | | |
| Recess 3  13:00:13:10 | □A sitting and chatting, reading or doing homework □B walk around (e.g., Go to toilet)  □C active play (indoor) □D active play (outdoor) | | | | | | | |
| Recess 4  13:40-13:50 | □A sitting and chatting, reading or doing homework □B walk around (e.g., Go to toilet)  □C active play (indoor) □D active play (outdoor) | | | | | | | |
| Recess 5  14:30-14:40 | □A sitting and chatting, reading or doing homework □B walk around (e.g., Go to toilet)  □C active play (indoor) □D active play (outdoor) | | | | | | | |
| Recess 6  15:25-15:35 | □A sitting and chatting, reading or doing homework □B walk around (e.g., Go to toilet)  □C active play (indoor) □D active play (outdoor) | | | | | | | |
| Recess 7  16:15-16:25 | □A sitting and chatting, reading or doing homework □B walk around (e.g., Go to toilet)  □C active play (indoor) □D active play (outdoor) | | | | | | | |
| leave school | Transportation | | □A cycling □B by bus □C walk □D Parents' transfer | | | | | |
| *Time block* | *Activity number*  *(According to page 2)* | | | *Self-feeling (according to page 1)* | | | | |
|  |  |  |  | *Sedentary Behavior* | | *Light* | *Moderate* | *Vigorous* |
| 17:05-17:30 |  | | |  | |  |  |  |
| 17:30-18:00 |  | | |  | |  |  |  |
| 18:00-18:30 |  | | |  | |  |  |  |
| 18:30-19:00 |  | | |  | |  |  |  |
| 19:00-19:30 |  | | |  | |  |  |  |
| 19:30-20:00 |  | | |  | |  |  |  |
| 20:00-20:30 |  | | |  | |  |  |  |
| 20:30-21:00 |  | | |  | |  |  |  |
| 21:00-21:30 |  | | |  | |  |  |  |
| 21:30-22:00 |  | | |  | |  |  |  |
| 22:00-22:30 |  | | |  | |  |  |  |
| 22:30-23:00 |  | | |  | |  |  |  |
| 23:00-23:30 |  | | |  | |  |  |  |
| 23:30-24:00 |  | | |  | |  |  |  |

**Weekend**

| *Time block* | *Activity number*  *(According to page 2)* | *Self-feeling (according to page 1)* | | | |
| --- | --- | --- | --- | --- | --- |
|  |  | *Sedentary Behavior* | *Light* | *Moderate* | *Vigorous* |
| 6：00-6：30 |  |  |  |  |  |
| 6：30-7：00 |  |  |  |  |  |
| 7：00-7：30 |  |  |  |  |  |
| 7：30-8：00 |  |  |  |  |  |
| 8：00-8：30 |  |  |  |  |  |
| 8：30-9：00 |  |  |  |  |  |
| 9：00-9：30 |  |  |  |  |  |
| 9：30-10：00 |  |  |  |  |  |
| 10：00-10：30 |  |  |  |  |  |
| 10：30-11：00 |  |  |  |  |  |
| 11：00-11：30 |  |  |  |  |  |
| 11：30-12：00 |  |  |  |  |  |
| 12：00-12：30 |  |  |  |  |  |
| 12：30-13：00 |  |  |  |  |  |
| 13：00-13：30 |  |  |  |  |  |
| 13：30-14：00 |  |  |  |  |  |
| 14：00-14：30 |  |  |  |  |  |
| 14：30-15：00 |  |  |  |  |  |
| 15：00-15：30 |  |  |  |  |  |
| 15：30-16：00 |  |  |  |  |  |
| 16：00-16：30 |  |  |  |  |  |
| 16：30-17：00 |  |  |  |  |  |
| 17：00-17：30 |  |  |  |  |  |
| 17：30-18：00 |  |  |  |  |  |
| 18：00-18：30 |  |  |  |  |  |
| 18：30-19：00 |  |  |  |  |  |
| 19：00-19：30 |  |  |  |  |  |
| 19：30-20：00 |  |  |  |  |  |
| 20：00-20：30 |  |  |  |  |  |
| 20：30-21：00 |  |  |  |  |  |
| 21：00-21：30 |  |  |  |  |  |
| 21：30-22：00 |  |  |  |  |  |
| 22：00-22：30 |  |  |  |  |  |
| 22：30-23：00 |  |  |  |  |  |
| 23：00-23：30 |  |  |  |  |  |
| 23：30-00：00 |  |  |  |  |  |
